# Sex-Specific Routes To Immune Senescence In *Drosophila melanogaster*

**DOI:** 10.1038/s41598-017-11021-6

**Published:** 2017-09-05

**Authors:** Marco Kubiak, Matthew C. Tinsley

**Affiliations:** Biological and Environmental Sciences, University of Stirling, Stirling, FK9 4LA United Kingdom

## Abstract

Animal immune systems change dramatically during the ageing process, often accompanied by major increases in pathogen susceptibility. However, the extent to which senescent elevations in infection mortality are causally driven by deteriorations in canonical systemic immune processes is unclear. We studied *Drosophila melanogaster* and compared the relative contributions of impaired systemic immune defences and deteriorating barrier defences to increased pathogen susceptibility in aged flies. To assess senescent changes in systemic immune response efficacy we injected one and four-week old flies with the entomopathogenic fungus *Beauveria bassiana* and studied subsequent mortality; whereas to include the role of barrier defences we infected flies by dusting the cuticle with fungal spores. We show that the processes underlying pathogen defence senescence differ between males and females. Both sexes became more susceptible to infection as they aged. However, we conclude that for males, this was principally due to deterioration in barrier defences, whereas for females systemic immune defence senescence was mainly responsible. We discuss the potential roles of sex-specific selection on the immune system and behavioural variation between males and females in driving these different senescent trends.

## Introduction

Advanced age is often accompanied by increased infection burden^1^. Whilst many immunological processes show patterns of senescence^2^, the extent to which changes in any individual immune parameter drive age-dependent elevations in infection susceptibility is often unclear. Much research focusses on senescence of conventional immune response pathways^3–6^. However, the contribution of changes in other factors to age-dependent elevations in infection rates, such as barrier defence efficacy, hygiene behaviours and parasite exposure patterns, are often overlooked. In this study, we used the model insect *Drosophila melanogaster* to investigate the relative contributions of age-dependent declines in systemic immune responses and ageing deterioration in barrier defences to overall senescence of pathogen defence.

For most animal species, strong sex-specific selection on reproductive strategies and other aspects of life history, including parasite resistance, means that males and females often differ greatly in immunity, lifespan and ageing^7, 8^. In humans, males have shorter lifespan and are generally found to suffer earlier immunosenescent degeneration than females^9^. Divergent life-histories in males and females might lead one to predict that different aspects of pathogen defence would show sex-specific changes during the ageing process^10, 11^. However, explicit comparisons of immunosenescent processes in males and females are rare outside humans (but see refs 12–14). We addressed this by investigating whether the relative rates of barrier defence senescence and systemic immune senescence differ between the two sexes in *D. melanogaster*.

In comparison to vertebrates, the patterns of age-dependent change in immune parameters and pathogen susceptibility in insects are less clearly established. Even for insects with strong applied relevance, such as the insect vectors of human disease, studies vary in whether they detect age-associated changes in infection susceptibility, as well as in the direction and the magnitude of these changes^15–18^. Senescent declines in immune function are reported in the worm *Caenorhabditis elegans*, the cricket *Gryllus assimillis* and the bumblebee *Bombus terrestris* ^19–21^. In the best-studied insect immunological model, *D. melanogaster*, the few studies on immunosenescence have produced diverse results^14, 22–24^. Lesser *et al*.^22^ found an age-associated *increase* in the rate of bacterial clearance from the haemocoel in nearly half of wildtype genotypes, whereas other genotypes showed either no change, or evidence of senescence. Ramsden *et al*.^25^ found no decline in ability to clear bacterial infection with age, but suggested that infection tolerance declines in older flies. The induction of humoral immune response genes following infection changes as flies age^24^, the ability of immune cells to phagocytose pathogens deteriorates^14, 26^ and in females, but not males, immune cell abundance in the blood declines^14^.

Here we investigate how the ability of *D. melanogaster* flies to defend against infection by the entomopathogenic fungus *Beauveria bassiana* declines with age. This fungus will naturally infect *D. melanogaster*: when spores contact the fly cuticle they germinate, penetrating this barrier defence before growing systemically in the haemocoel^27^. It is also possible to inject *B. bassiana* spores directly into the fly, circumventing cuticle defences to test the efficacy of systemic immune processes. We assessed age-dependent changes in fly mortality following these two infection routes, testing the relative contribution of declines in barrier defences and systemic immune responses to overall senescence of pathogen protection. Our work demonstrates that the relative importance of senescence in these two pathogen defence systems differs strongly between males and females.

## Results

To quantify the relative contribution of declines in barrier defences and systemic immune responses to overall senescence of pathogen defence we monitored survival of 3,271 flies, either infected with the fungal pathogen *B. bassiana* or subjected to a control treatment (188 infected vials and 186 uninfected controls; mean flies per vial 9.36). Flies were 1 or 4 weeks old and were either infected through haemocoelic injection or by cuticle exposure. Averaging across both sexes and infection routes we found a significant decrease in survival probability of pathogen-exposed flies as they aged (${\chi }_{1}^{2}$ = 107.98, p < 0.001): 59.68% (±2.95%) of 1 week old flies survived until our assay cut-off time, whereas for 4-week-old flies this fell to 35.18% survival (±2.79%). However, this pattern of senescent decline in pathogen defence differed strongly between males and females depending on infection route.

**Figure 1:**
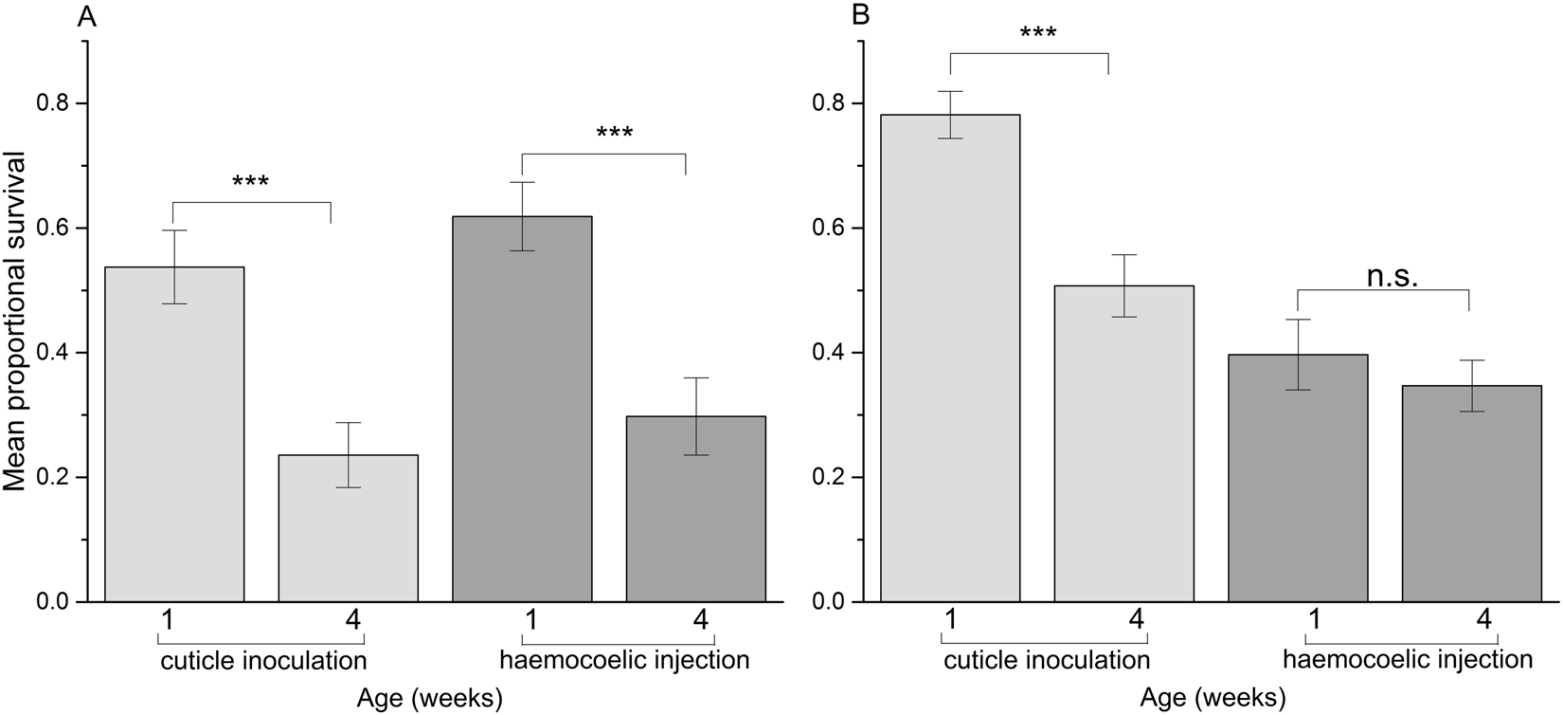
Mean proportional survival of 1 and 4-week-old flies after cuticle inoculation or haemocoelic injection with *Beauveria bassiana* spores. To make both infection treatments comparable, we selected data from the timepoint where overall mean proportional survival was closest to 50% for each treatment route: day 7 for cuticle inoculation and day 5 for haemocoelic injection (see methods). (**A**) Female flies: both infection treatments showed the same age-dependent decline in survivorship. (**B**) male flies: cuticle inoculated flies showed a strong decrease in survival while aging, whereas pathogen injected flies showed only a minimal change in survivorship. Uninfected control flies showed minimal mortality during this time frame (see text). Error bars show ± SE; significance of comparisons shown by ***P < 0.001.

In female flies, survival after pathogen inoculation decreased from 53.74% (±5.09%) to 23.58% (±5.21%) (${\chi }_{1}^{2}$ = 53.66, p < 0.001) between 1 and 4 weeks of age in the cuticle exposure treatment; survival declined by a similar magnitude (a fall of 32.08% ± 5.93%) in the pathogen injection treatment (${\chi }_{1}^{2}$ = 31.78, p < 0.001) (Fig. 1A). This pattern of age-dependent decline in pathogen defence did not differ between the two infection routes for females (age by infection route interaction: ${\chi }_{1}^{2}$ = 0.48, p = 0.49). In contrast, for males there was only a marginal and non-significant decrease in survival of 5.00% (±5.93%) between 1 and 4 weeks of age in the haemocoelic injection treatment (${\chi }_{1}^{2}$ = 0.41, p = 0.52); whereas survival declined from 78.15% (±3.79%) to 50.72% (±4.99%) between 1 and 4 weeks of age in flies infected by cuticle exposure (${\chi }_{1}^{2}$ = 50.46, p < 0.001) (Fig. 1B). For males, the difference between these two infection routes in the age-dependent change in pathogen susceptibility was strongly significant (age by infection route interaction: ${\chi }_{1}^{2}$ = 17.12, p < 0.001). This major difference between males and females in the relative rate of pathogen defence senescence in the two infection routes was supported by a significant three-way interaction between sex, age and infection route (${\chi }_{1}^{2}$ = 7.33, p = 0.007).

For 1 week old flies, males survived cuticle exposure to *B. bassiana* spores until the assay cut-off time significantly better than females (${\chi }_{1}^{2}$ = 41.93, p < 0.001, Fig. 1), a trend that was still significant in 4 week old individuals (${\chi }_{1}^{2}$ = 40.86, p < 0.001). In contrast, for the spore injection assay, survival of females was higher than males for 1 week old individuals (${\chi }_{1}^{2}$ = 19.37, p < 0.001, Fig. 1), but for this infection route there was no significant difference between the sexes by four weeks of age (${\chi }_{1}^{2}$ = 0.32, p = 0.57, Fig. 1).

**Figure 2:**
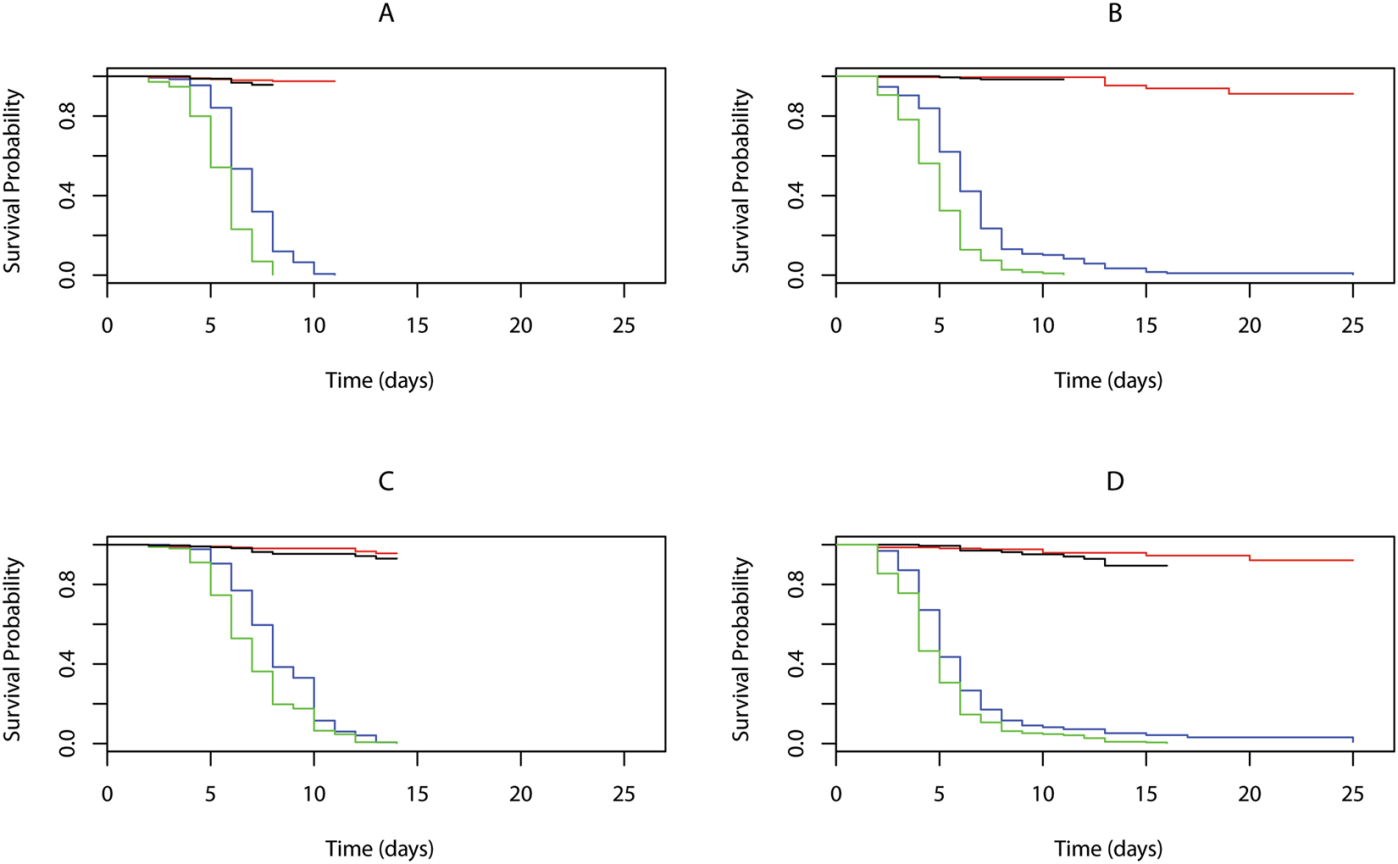
Survival curves for female and male *D. melanogaster* after cuticle inoculation or haemocoelic injection with *B. bassiana* spores. (**A**) Female survival after cuticle inoculation, (**B**) Female survival after haemocoelic injection, (**C**) Male survival after cuticle inoculation, (**D**) Male survival after haemocoelic injection. Coloured lines show: blue (age 1 week) and green (age 4 weeks) survival of flies that received a pathogen infection treatment, whereas red (age 1 week) and black (age 4 weeks) show survival of uninfected control flies. The comparisons between 1 and 4 week flies infected with spores were significant for A, B & C, but not for D. Control observations ceased at the time point when no infected flies for the corresponding treatment category remained alive, therefore the time-course of observations for assays A–D varies.

Rather than solely studying the outcome of infection as the proportion of flies that survived until a single time point, we also used survival analysis to investigate mortality risk variation across the whole dataset for the full experiment time course (Fig. 2), testing whether the entire pattern of post-infection mortality supported these trends. This alternative analysis approach verified that averaging across all infected treatments older flies died at a faster rate post-infection than younger flies (${\chi }_{1}^{2}$ = 508.56, p < 0.001), that for females there was no difference in senescence of pathogen defence between the two infection routes (age by infection route interaction: ${\chi }_{1}^{2}$ = 2.10 p = 0.15), whilst for males, age dependent deterioration in pathogen defence occurred more strongly in cuticle-exposed flies than in injected flies (age by infection route interaction: ${\chi }_{1}^{2}$ = 4.00, p = 0.045).

To demonstrate that this senescence in post-infection survival was specifically due to declines in pathogen defence, we investigated these same age-dependent trends in the control treatments, where flies were not exposed to the *B. bassiana* pathogen. Survival of these controls until our assay cut-off time was very high (Fig. 2). Averaging across both sexes and infection routes, mean survival for 1 week old flies until the assay cut-off time was 98.55% (±0.44%), compared to 97.88% (±0.42%) for 4-week-old flies, a difference that was not significant (${\chi }_{1}^{2}$ = 0.55, p = 0.46). Further analyses with both, linear-mixed-effect models and coxme survival models found no significant survival differences between the sexes, age groups or infection routes within the control flies.

## Discussion

In this study, we demonstrate that pathogen defence senescence occurs by different processes in male and female *D. melanogaster*. We conclude that whilst flies of both sexes become more susceptible to fungal infection as they age, for males this occurs principally due to deterioration in barrier defences, whereas for females it is most likely that systemic immune senescence is mainly responsible.

Our experiments assessed overall senescence of pathogen defence by comparing the infection susceptibility of 1 and 4-week-old flies dusted with entomopathogenic fungal spores. In parallel we isolated the contribution of age-dependent changes in systemic immune responses to this senescent process by directly injecting fungal spores into the haemocoel, circumventing cuticle barrier defences. For male flies, pathogen-induced mortality did not increase during ageing when we injected spores into the haemocoel, but there was a strong age-associated increase in infection susceptibility when we dusted spores onto the cuticle. We conclude that, for males, whilst the efficacy of systemic defence changes little, ageing leads to deterioration of the cuticle barrier defences, meaning that a larger pathogen dose penetrates into the haemocoel and overwhelms the immune response in older flies. In contrast, female flies showed the same magnitude of age-associated decline in survivorship in both these infection assays. In our spore dusting assay, systemic and barrier defences combine to influence mortality, but only systemic defences are relevant to the injection assay. Therefore, for females, we conclude that age-related changes in the systemic immune response are probably sufficient to explain senescence of infection defence, with no additional contribution from senescent changes in barrier defences.

Previous research on invertebrate immunosenescence has focussed on studying changes in systemic immune responses, demonstrating ageing-associated alterations in antimicrobial peptide expression, phenoloxidase activity, microbial phagocytosis by haemocytes and haemocyte abundance^14, 20, 21, 24, 28, 29^. Whilst invertebrate research on how the senescence of systemic immunity differs between males and females is rare, Mackenzie *et al*.^14^ found that the density of haemocytes circulating in fly haemolymph declined with age in female flies, but not in males. If the density of haemocytes in the blood is a major determinant of successful pathogen defence against *B. bassiana*, it is possible that this sex-specific senescent change in cellular immunity could explain our current observation that systemic pathogen defence senesces in females but not males. We know of no existing studies investigating how senescence influences barrier defence efficacy in invertebrates. However, the external epithelia, such as the trachea, reproductive tract, and gut do all mount vigorous local immune responses, including constitutive antimicrobial peptide synthesis^30^ that might alter with age. If age-dependent changes in these responses do indeed occur and contribute to pathogen defence senescence, then our data suggest the hypothesis that senescent decline in epithelial immune response gene expression in males might be stronger than in females. The age-dependent deterioration of barrier defences in males might instead be due to processes that are not classically associated with the immune response. Flies vigorously clean themselves after being dusted by fungal spores^31^. Hygienic behaviours, such as spore removal, might senesce faster in males than in females meaning that elderly males become infected by a larger fungal burden. Alternatively, because the pathogenicity of entomopathogenic fungal spores depends on their ability to metabolise specific hydrocarbons on the cuticle^32^ and cuticle hydrocarbon profiles change with age in *D. melanogaster* ^33^ we speculate that sex-differences in cuticle hydrocarbon senescence might potentially contribute to driving senescence of barrier defences in male flies.

Differences between individuals in their likelihood of death following infection may be either because of variation in infection resistance or infection tolerance^34^. We did not investigate this in the current study. However, the results of a study by Ramsden *et al*.^25^ investigating senescence of the ability of *D. melanogaster* to defend against bacteria injected into the haemocoel indicated that elevated pathogen-induced mortality in old flies was not driven by impaired resistance, it was therefore probably driven by declines in infection tolerance. The importance of declines in tolerance and resistance to explaining senescence of pathogen defence may differ between males and females. If barrier defences principally operate by preventing the penetration of pathogens, then the senescence we observed in males may be driven by a decline in infection resistance. Although the systemic pathogen defence senescence we observed in females could be driven by either process, if the findings of Ramsden *et al*.^25^ can be generalised to our study, perhaps elderly females die because they cannot tolerate the same infection burden as young individuals. We note that a small proportion (<0.5%) of 1 week old individuals of both sexes that received *B. bassiana* spore injections survived for a protracted period after infection before dying. We previously showed that flies can survive for at least 1 month whist still incubating a live *B. bassiana* infection^35^. We do not know whether these individuals develop a disease-tolerant state, or if they resit infection without being able to eliminate it. It would be fruitful for future studies to determine the importance of declining resistance and tolerance in the senescent effects we have observed. In our haemoceolic injection treatment all flies received injections into the thorax. The location of the injection site has been shown to influence the magnitude of infection-induced mortality^36^; it would be interesting to investigate if the magnitude of the senescent and sex-specific effects we uncovered are influenced by infection site.

The ultimate reason for this difference in the pattern of pathogen defence senescence between males and females may lie either in divergent behaviour or differential life history selection between the two sexes. Our data show that for young flies, males are more susceptible to *B. bassiana* than females when they are dusted with spores; dimorphism that has been reported previously^31^. However, interestingly, males were more susceptible than females when spores were injected into the haemocoel. This could indicate that young males invest relatively more than females in barrier defences, but less in systemic immunity. If barrier defence efficacy against pathogens is determined mostly by cuticle integrity, males may have been selected to have more resilient cuticle structure to defend against aggressive encounters with other males^37^. Our conclusion that males suffer pathogen defence senescence because of barrier defence deterioration could be explained if repeated wounding during a lifetime of aggressive male-male encounters means that spores can penetrate the cuticle of older flies more efficiently. Furthermore, if the level of cuticle pathogen defence is principally governed by more dynamic immunological processes, age-dependent declines in male aggression might selectively favour adaptive down regulation of cuticle defence mechanisms. Indeed, previous work demonstrated that male aggression increases significantly from 1 to 4 days of age but then exhibits a non-significant decline between 4 of 7 days of age^38^; it would be interesting to investigate later life timepoints. Lower investment in costly systemic pathogen defence by males is consistent with much of the sizeable body of literature on sex biased parasitism^12, 39^. If females do invest more than males in systemic immune defences when they are young, it may be that females experience a larger senescent decline in these immune responses due to a higher starting point at young ages.

Whilst our infection protocols were identical for males and females, females are larger than males, which may have influenced the impacts of our standardised spore injection and cuticle exposure treatments. Relative to their smaller bodyweight, males will have received more spores than females in the haemocoel injection treatment. Similarly, whilst larger female body size means that more spores may have adhered to the cuticle of females during the dusting treatment, the greater surface area to volume ratio of males means that they probably received a higher inoculum relative to bodyweight during the cuticle exposure assay as well. Nevertheless, Taylor and Kimbrell^31^ used a *B. bassiana* dusting assay to demonstrate that mortality differences between male and female flies following spore exposure could not be explained simply by dose differences between the sexes. Whether or not these relative differences in pathogen dose influence sex differences in infection mortality in our study, our study conclusions are principally focussed on the degree of age-dependent infection susceptibly change that occurs within each sex, which is unlikely to have been influenced strongly by body-size effects.

The extent to which our finding of sex-specific routes to immune senescence can be generalised to other pathogens and to other animal species will need to be assessed. Nevertheless, there are some parallels for our observation of age-related barrier defence impairment in medical science, where research on vertebrate models of ageing demonstrates that cellular senescence and impaired repair mechanisms in the respiratory epithelia of elderly individuals may predispose them to infection^40, 41^. Furthermore, the impacts of ageing on human traits such as senescence of infection resistance and age-dependent declines in vaccine responsiveness differ strongly in males and females^42^. Understanding sex-specific routes to immune senescence in insects may be of strong applied relevance. Knowledge of the determinants of infection susceptibility to entomopathogenic fungi, such as *B. bassiana* that we studied, is important because these pathogens hold promise for the control of the mosquito vectors of malaria and other human diseases^43^. Furthermore, in the context of the insect vectors of human infections, the pathogen susceptibility of older insects is especially important because it is predominantly older female mosquitos that are responsible for malaria transmission^44^. Our results demonstrating asymmetries in pathogen defence between males and females are of particular relevance to insect biological control strategies that use autodissemination techniques, in which spore-inoculated males are exploited to transmit infection to females of vector or pest populations^45^.

The *Drosophila* immunosenescence literature currently contains a variety of apparent inconsistencies, during ageing: immune gene transcript levels generally become elevated^24, 29^; pathogen clearance abilities do not alter consistently, but directions of age-dependent change vary across genotypes^22, 46^; phagocytosis rates by immune cells go down^14, 26^. Surprisingly, only one previous study, working with *E. coli*, has investigated senescent changes in mortality following infection^25^. Our current work adds a second case study which demonstrates that *D. melanogaster* flies, as do humans, suffer greater infection-induced mortality as they age. Previous mechanistic studies of immunosenescence in *Drosophila* have perhaps been hampered because there is limited understanding of which age-dependent changes in the immune system (or other physiological systems) are causally responsible for elevated pathogen susceptibility in elderly flies. One promising approach for the future will be to assess variation in the age-dependent rate of infection defence deterioration across a group of wild-type genotypes, then attempt to correlate these differences with variation in a range of potentially causal immune system parameters^46^. Our current study emphasises that these correlations may not be the same in the two sexes because the processes underlying pathogen defence senescence differ. Our work illustrates the overarching principle that patterns of immune senescence can vary strongly between males and females: the causes and consequences of this deserve further investigation.

## Materials and Methods

### Fly culturing

We reared flies at a constant density to the age of 1 and 4 weeks following the methods of Mackenzie *et al*.^14^. We placed Samarkand genotype *D. melanogaster* adults (from Bloomington Stock Centre) into a laying cage and collected eggs for 24 h on apple juice agar plates seeded with yeast. Following Clancy & Kennington^47^, eggs were washed from the plate with PBS buffer, then a 13 µl volume of packed eggs was added to the food medium in fly bottles with a pipette. When adult flies emerged, they were transferred to age in 11 litre fly cages at a 1:1 sex ratio with 200 flies per cage. Every two days cages were provided with a new petri dish of fly food and a vial of water. Cages were set up weekly in groups of three: two cages to supply known-age flies to experiments and a third to provide flies in order to maintain the other two cages at constant density by replacing flies that died. Flies for replacement were moved between cages using an electronic pooter; the mean number of total flies replaced in the lead up to using each 1-week and 4-week cage was 3.0 (±0.2) and 22.0 (±0.7) flies respectively. Cages were taken when aged 1 week, or when aged 4 weeks, to supply flies to either the cuticle inoculation or the pathogen injection experimental assays. All flies were fed Lewis *Drosophila* medium^48^ and maintained at 25 °C, 12 h L/D throughout.

### Pathogen Infection

The isolate of the pathogen *B. bassiana* was derived from a previous experiment in which strain 193–825 (IMI 391510) was passaged for one generation through a genetically diverse population of *D. yakuba* ^35^. The fungus was grown by plating onto potato dextrose agar containing chloramphenicol antibiotic (5 × 10^−5^ g ml^−1^); plates were incubated at 25 °C in the dark, then dried for five days at room temperature. After sporulation, spores were scraped off the plates, pooled and stored at 4 °C in tubes containing silica gel to protect against moisture.

To infect flies via cuticle inoculation dry fungal spores were mixed 1% w/w with agar powder, then this mixture was scattered into a 9 cm petri dish until the bottom was thinly covered. CO_2_-anaestethized flies in single-sex batches of 10 were shaken in the petri dish until flies were uniformly covered, then put in vials with standard Lewis food medium that did not contain nipagin (to exclude the possible influence of this anti-fungal agent on mortality patterns). The agar-spore mixture was replenished regularly. An equal number of flies also experienced a control procedure, where flies received the same dusting treatment, but with agar only. To infect flies systemically, circumventing the cuticle defences, we injected the pathogen. For this assay, fungal spores were suspended in oil (87.5% Shellsol T: 12.5% Ondina EL) at 1.3 × 10^8^ spores ml^−1^ ^35^; single-sex 10-fly groups were CO_2_ anaesthetized and pricked in the thorax with a stainless needle (AgnTho’s minutien pins No. 26002–20, 0.2 mm diameter) that had been dipped into the fungal spore suspension. To make the injection depth consistent between replicates the needle was always inserted into the cuticle up to the shoulder of the needle’s tapering point. Flies were then transferred to fresh nipagin-free food vials. Control flies received identical oil-only injections. A small number of flies that died within 2 hours were not considered to have died from the pathogen and excluded from analysis. For both the cuticle inoculation and injection experiments, vials were subsequently inspected at the same time daily and dead flies counted; flies were changed to new vials every 2 days, before any of the dead cadavers in the infected treatment sporulated. The overall study was split into five separate blocks for the systemic infection and three separate blocks for the cuticular inoculation treatments.

### Statistics

The time course of mortality post-infection differed slightly between the two infection routes. Therefore, to compare the age-dependent mortality trends we studied the probability of fly survival until a cut off time closest to when 50% of all infected flies had died in each assay: day 5 and day 7 for haemocoel injection and cuticle exposure treatments respectively. We first focussed on the data from flies in the infected treatments. We used the glmer function from lme4^49^ in R (version 3.2.3)^50^ and built linear mixed-effect models using sex, age, infection route and all interactions up to 3-way as fixed factors and experimental block as a random factor. Fly vial (each containing approximately 10 flies) was the unit of replication. Models had a binomial error structure and used a two-vector response containing the number of flies that were dead and alive in each vial. Models were sequentially simplified and the significance of terms of interest was tested using Likelihood Ratio tests for model comparisons. To demonstrate that mortality variation resulted from pathogen infection, we verified that the treatment group differences amongst the infected flies were not also present in the control dataset, and further tested for the presence of higher level interactions with the term ‘infection status’ across the entire data set. Lastly, we took an independent analytical approach that used the whole dataset and employed survival analysis to assess the temporal pattern of post-infection mortality using the coxme package^51^ with the fixed and random effect structure described above. We verified the assumptions of our statistical models by inspecting diagnostics plots. We checked that hazard rates of the treatment categories in our survival analysis were proportional by re-running survival models using the coxph function from the R package Survival^52^ and then testing proportionality using the cox.zph function. Relevant means and differences were calculated from raw data and are stated in the text with their standard errors.
